# 1-Benzyl-5-ethyl-5-hy­droxy-1*H*-pyrrol-2(5*H*)-one

**DOI:** 10.1107/S1600536813016887

**Published:** 2013-06-22

**Authors:** Yan-Jiao Gao, Yu-Huang Wang, Jian-Liang Ye

**Affiliations:** a The Key Laboratory for Chemical Biology of Fujian Province, College of Chemistry and Chemical Engineering, Xiamen University, Xiamen, Fujian 361005, People’s Republic of China

## Abstract

The title compound, C_13_H_15_NO_2_, was obtained as a by-product in the Grignard reaction of malimide. The dihedral angle between the five-memebred ring (r.m.s. deviation = 0.005 Å) and the benzene ring is 67.20 (14)°. The benzene ring and the ethyl chain lie to the same side of the five-membered ring. In the crystal, mol­ecules are linked by O—H⋯O hydrogen bonds, generating *C*(6) chains propagating in [010].

## Related literature

For background to the Grignard reaction of malimide, see: Huang (2006 ▶); He *et al.* (2003 ▶). For related structures, see: Goh *et al.* (2007 ▶); Ma & Xie (2002 ▶).
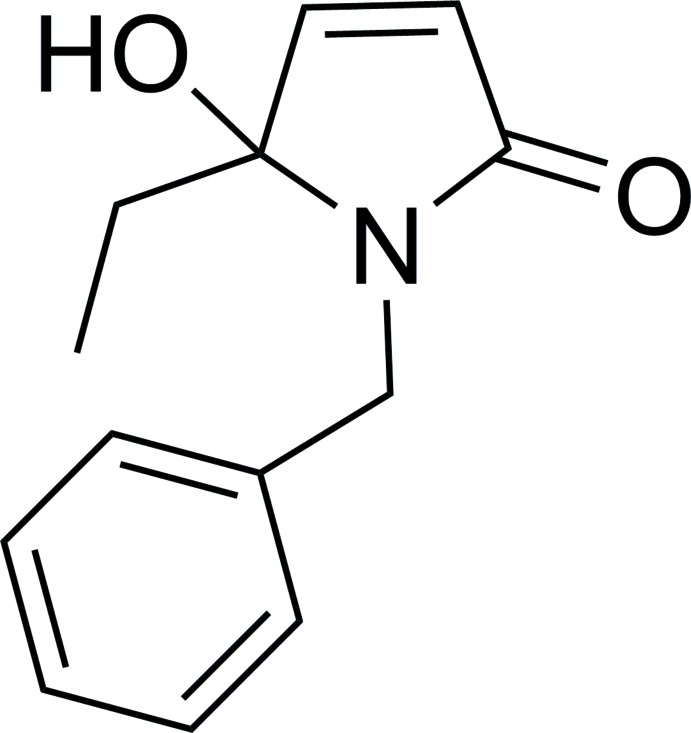


## Experimental

### 

#### Crystal data

C_13_H_15_NO_2_
*M* *_r_* = 215.27
Monoclinic, 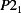

*a* = 7.0399 (14) Å
*b* = 7.1795 (14) Å
*c* = 11.817 (2) Å
β = 102.72 (3)°
*V* = 582.6 (2) Å^3^
*Z* = 2
Mo *K*α radiation
μ = 0.08 mm^−1^
*T* = 173 K
0.3 × 0.2 × 0.2 mm

#### Data collection

Oxford Diffraction Xcalibur (Sapphire3, Gemini ultra) diffractometer
Absorption correction: multi-scan (*CrysAlis PRO*; Oxford Diffraction, 2010 ▶) *T*_min_ = 0.980, *T*_max_ = 0.983
3363 measured reflections
1947 independent reflections
1811 reflections with *I* > 2σ(*I*)
*R*_int_ = 0.054

#### Refinement

*R*[*F* ^2^ > 2σ(*F* ^2^)] = 0.044
*wR*(*F* ^2^) = 0.145
*S* = 1.13
1947 reflections
145 parameters
1 restraint
H-atom parameters constrained
Δρ_max_ = 0.25 e Å^−3^
Δρ_min_ = −0.32 e Å^−3^

### 

Data collection: *CrysAlis PRO* (Oxford Diffraction, 2010 ▶); cell refinement: *CrysAlis PRO*; data reduction: *CrysAlis PRO*; program(s) used to solve structure: *SHELXTL* (Sheldrick, 2008 ▶); program(s) used to refine structure: *SHELXTL*; molecular graphics: *SHELXTL*; software used to prepare material for publication: *SHELXTL*.

## Supplementary Material

Crystal structure: contains datablock(s) I, global. DOI: 10.1107/S1600536813016887/hb7096sup1.cif

Structure factors: contains datablock(s) I. DOI: 10.1107/S1600536813016887/hb7096Isup2.hkl

Supplementary material file. DOI: 10.1107/S1600536813016887/hb7096Isup3.cml

Additional supplementary materials:  crystallographic information; 3D view; checkCIF report

## Figures and Tables

**Table 1:** Hydrogen-bond geometry (Å, °)

*D*—H⋯*A*	*D*—H	H⋯*A*	*D*⋯*A*	*D*—H⋯*A*
O2—H2*A*⋯O1^i^	0.82	1.95	2.772 (3)	176

Symmetry code: (i) 
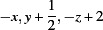
.

**Fig. 1.**
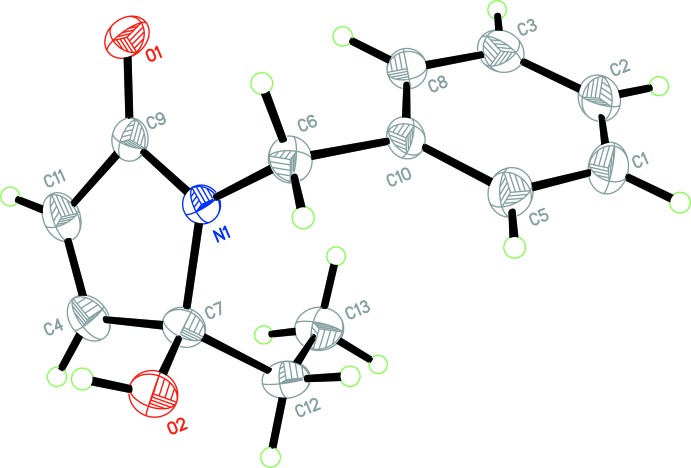
The molecular structure of the title compound showing 50% probability displacement ellipsoids.

**Fig. 2.**
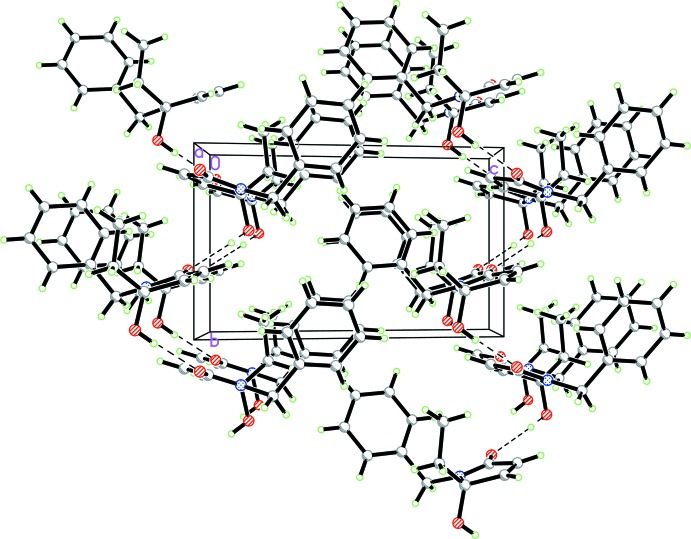
The packing of the molecules, viewed down the a axis. O—H···O hydrogen bond interactions are shown as dashed lines.

## supplementary crystallographic information

### Comment

Using Grignard reagents as the nucleophiles allows a flexible introduction of diverse side chains at the C-2 carbonyl of malimides (Huang, 2006). In addition, Grignard reagents are essentially strong bases, so the addition of a Grignard reagent to a malimide provided an unexpected 3-alkoxy group elimination product *rac*-1-benzyl-5-methyl-1*H*-pyrrol-2(5*H*)-one (He *et al.*, 2003). Recently a new addition-elimination product, *rac*-1-benzyl-5-ethyl-5-hydroxy-1*H*-pyrrol-2(5*H*)-one, was found in the Grignard addition reaction. Here we report the structure of the title compound.

In γ-lactam ring the vinyl carbon atoms remain almost coplanar with the amide moiety [r.m.s. 0.0006 Å], which are agreement with the similar compounds (Goh *et al.*, 2007; Ma & Xie, 2002). In the crystal, the molecules are linked by O—H···O hydrogen bonds between the hydroxyl group and the oxygen atom of the carbonyl group.

### Experimental

To a stirred solution of (*S*)-*N*,*O*-benzyl-malimide ((*S*)-1-benzyl-3-(benzyloxy)pyrrolidine-2,5-dione) (2 mmol) in anhydrous CH_2_Cl_2_ (20 ml) was added dropwise EtMgBr (4 mmol) in diethyl ether at -20 °C under nitrogen atmosphere. The mixture was stirred at -20 °C for 1 h and then quenched by adding a saturated aqueous solution of NH_4_Cl. The mixture was extracted with CH_2_Cl_2_ (4 × 10 ml). The combined extracts were washed with brine, dried over Na_2_SO4, concentrated under reduced pressure. The residue was purified by flash chromatography (eluent: EtOAc/PE = 1: 2; then 2: 1), provided a mixture of diastereomers (4*S*)-1-benzyl-4-(benzyloxy)-5-ethyl-5-hydroxypyrrolidin-2-one as major products (white crystals, yield 85%) and the title compound as minor product (colourless crystals, yield 10%). Colourless pillars of the tiltle compound were obtained by slow evaporation of a mixture of *n*-hexane/ethyl acetate solution.

### Refinement

The hydrogen atoms were positioned geometrically, with C—H = 0.93, 0.98, 0.97 and 0.96 Å for phenyl, methine, methylene and methyl H atoms, respectively, and were included in the refinement in the riding model approximation. The displacement parameters of methyl H atoms were set to 1.5*U*_eq_(C), while those of other H atoms were set to 1.2*U*_eq_(C). In the absence of significant anomalous scattering effects the absolute structure of the chosen crystal was indeterminate.

### Crystal data

**Table:**

C_13_H_15_NO_2_	*F*(000) = 230
*M**_r_* = 215.27	*D*_x_ = 1.227 Mg m^−^^3^
Monoclinic, *P*2_1_	Mo *K*α radiation, λ = 0.71073 Å
*a* = 7.0399 (14) Å	Cell parameters from 2119 reflections
*b* = 7.1795 (14) Å	θ = 3.0–27.0°
*c* = 11.817 (2) Å	µ = 0.08 mm^−^^1^
β = 102.72 (3)°	*T* = 173 K
*V* = 582.6 (2) Å^3^	Pillar, colourless
*Z* = 2	0.3 × 0.2 × 0.2 mm

### Data collection

**Table:**

Oxford Diffraction Xcalibur (Sapphire3, Gemini ultra) diffractometer	1947 independent reflections
Radiation source: Enhance (Mo) X-ray Source	1811 reflections with *I* > 2σ(*I*)
Graphite monochromator	*R*_int_ = 0.054
Detector resolution: 16.1903 pixels mm^-1^	θ_max_ = 27.0°, θ_min_ = 3.0°
phi and ω scans	*h* = −8→8
Absorption correction: multi-scan (*CrysAlis PRO*; Oxford Diffraction, 2010)	*k* = −9→9
*T*_min_ = 0.980, *T*_max_ = 0.983	*l* = −14→15
3363 measured reflections	

### Refinement

**Table:**

Refinement on *F*^2^	Primary atom site location: structure-invariant direct methods
Least-squares matrix: full	Secondary atom site location: difference Fourier map
*R*[*F*^2^ > 2σ(*F*^2^)] = 0.044	Hydrogen site location: inferred from neighbouring sites
*wR*(*F*^2^) = 0.145	H-atom parameters constrained
*S* = 1.13	*w* = 1/[σ^2^(*F*_o_^2^) + (0.0893*P*)^2^ + 0.0423*P*] where *P* = (*F*_o_^2^ + 2*F*_c_^2^)/3
1947 reflections	(Δ/σ)_max_ < 0.001
145 parameters	Δρ_max_ = 0.25 e Å^−^^3^
1 restraint	Δρ_min_ = −0.32 e Å^−^^3^

### Special details

**Table:**

Experimental. Absorption correction: CrysAlisPro, Oxford Diffraction Ltd., Version 1.171.34.44. Empirical absorption correction using spherical harmonics, implemented in SCALE3 ABSPACK scaling algorithm.
Geometry. All e.s.d.'s (except the e.s.d. in the dihedral angle between two l.s. planes) are estimated using the full covariance matrix. The cell e.s.d.'s are taken into account individually in the estimation of e.s.d.'s in distances, angles and torsion angles; correlations between e.s.d.'s in cell parameters are only used when they are defined by crystal symmetry. An approximate (isotropic) treatment of cell e.s.d.'s is used for estimating e.s.d.'s involving l.s. planes.
Refinement. Refinement of *F*^2^ against ALL reflections. The weighted *R*-factor *wR* and goodness of fit *S* are based on *F*^2^, conventional *R*-factors *R* are based on *F*, with *F* set to zero for negative *F*^2^. The threshold expression of *F*^2^ > σ(*F*^2^) is used only for calculating *R*-factors(gt) *etc*. and is not relevant to the choice of reflections for refinement. *R*-factors based on *F*^2^ are statistically about twice as large as those based on *F*, and *R*- factors based on ALL data will be even larger.

### Fractional atomic coordinates and isotropic or equivalent isotropic displacement parameters (Å^2^)

**Table:**

	*x*	*y*	*z*	*U*_iso_*/*U*_eq_	
O1	−0.2177 (3)	0.6481 (3)	0.96950 (15)	0.0294 (4)	
N1	−0.0106 (3)	0.7331 (3)	0.85306 (15)	0.0214 (4)	
C1	−0.2478 (4)	0.4825 (5)	0.4747 (2)	0.0383 (7)	
H1A	−0.2359	0.4924	0.3981	0.046*	
O2	0.2472 (3)	0.9389 (2)	0.83792 (15)	0.0294 (4)	
H2A	0.2397	1.0049	0.8934	0.044*	
C2	−0.3230 (4)	0.3208 (5)	0.5114 (2)	0.0403 (7)	
H2B	−0.36	0.2222	0.4602	0.048*	
C3	−0.3426 (4)	0.3079 (5)	0.6254 (2)	0.0352 (6)	
H3A	−0.3927	0.2	0.6511	0.042*	
C4	0.2774 (4)	0.6967 (4)	0.9869 (2)	0.0291 (6)	
H4A	0.4086	0.6937	1.0235	0.035*	
C5	−0.1894 (4)	0.6312 (4)	0.5507 (2)	0.0325 (6)	
H5A	−0.1362	0.738	0.5255	0.039*	
C6	−0.1548 (4)	0.7814 (4)	0.74846 (19)	0.0254 (5)	
H6A	−0.1033	0.8808	0.7084	0.03*	
H6B	−0.2707	0.8281	0.7706	0.03*	
C7	0.2009 (4)	0.7521 (3)	0.8611 (2)	0.0241 (5)	
C8	−0.2875 (4)	0.4557 (4)	0.7009 (2)	0.0283 (6)	
H8A	−0.3016	0.4459	0.7771	0.034*	
C9	−0.0541 (3)	0.6758 (3)	0.95313 (19)	0.0225 (5)	
C10	−0.2118 (3)	0.6177 (4)	0.66498 (19)	0.0252 (5)	
C11	0.1357 (4)	0.6535 (4)	1.0372 (2)	0.0300 (6)	
H11A	0.151	0.6149	1.1138	0.036*	
C12	0.2726 (4)	0.6284 (4)	0.7756 (2)	0.0286 (6)	
H12A	0.4104	0.652	0.7826	0.034*	
H12B	0.2061	0.6643	0.6978	0.034*	
C13	0.2442 (4)	0.4196 (4)	0.7893 (2)	0.0335 (6)	
H13A	0.2931	0.3535	0.7311	0.05*	
H13B	0.1081	0.3932	0.7807	0.05*	
H13C	0.3136	0.3806	0.8649	0.05*	

### Atomic displacement parameters (Å^2^)

**Table:**

	*U*^11^	*U*^22^	*U*^33^	*U*^12^	*U*^13^	*U*^23^
O1	0.0328 (9)	0.0290 (11)	0.0303 (9)	−0.0023 (8)	0.0153 (7)	−0.0011 (8)
N1	0.0209 (9)	0.0227 (10)	0.0203 (8)	0.0000 (8)	0.0035 (7)	−0.0002 (8)
C1	0.0360 (14)	0.053 (2)	0.0254 (12)	0.0033 (15)	0.0061 (10)	−0.0087 (13)
O2	0.0358 (10)	0.0214 (10)	0.0325 (9)	−0.0076 (8)	0.0104 (7)	0.0009 (7)
C2	0.0317 (14)	0.052 (2)	0.0370 (14)	−0.0020 (14)	0.0067 (11)	−0.0215 (14)
C3	0.0295 (14)	0.0360 (16)	0.0398 (14)	−0.0060 (12)	0.0068 (11)	−0.0067 (12)
C4	0.0289 (12)	0.0263 (14)	0.0283 (11)	−0.0024 (11)	−0.0020 (9)	0.0022 (10)
C5	0.0311 (13)	0.0398 (16)	0.0260 (12)	0.0042 (12)	0.0049 (10)	0.0011 (12)
C6	0.0249 (12)	0.0254 (13)	0.0242 (11)	0.0054 (10)	0.0017 (9)	0.0016 (10)
C7	0.0224 (11)	0.0218 (13)	0.0274 (11)	−0.0005 (10)	0.0039 (8)	0.0017 (10)
C8	0.0237 (11)	0.0339 (16)	0.0268 (11)	0.0028 (11)	0.0047 (8)	−0.0044 (11)
C9	0.0300 (12)	0.0150 (11)	0.0230 (10)	−0.0016 (10)	0.0069 (8)	−0.0021 (9)
C10	0.0196 (10)	0.0320 (14)	0.0222 (10)	0.0058 (11)	0.0004 (8)	−0.0033 (10)
C11	0.0363 (13)	0.0278 (14)	0.0225 (11)	−0.0053 (12)	−0.0008 (9)	0.0013 (10)
C12	0.0244 (12)	0.0275 (14)	0.0362 (13)	0.0000 (10)	0.0118 (9)	0.0008 (11)
C13	0.0310 (14)	0.0260 (13)	0.0449 (14)	0.0059 (12)	0.0114 (11)	−0.0022 (12)

### Geometric parameters (Å, º)

**Table:**

O1—C9	1.225 (3)	C5—C10	1.397 (3)
N1—C9	1.349 (3)	C5—H5A	0.93
N1—C6	1.458 (3)	C6—C10	1.530 (3)
N1—C7	1.477 (3)	C6—H6A	0.97
C1—C2	1.385 (5)	C6—H6B	0.97
C1—C5	1.397 (4)	C7—C12	1.513 (4)
C1—H1A	0.93	C8—C10	1.384 (4)
O2—C7	1.421 (3)	C8—H8A	0.93
O2—H2A	0.82	C9—C11	1.488 (3)
C2—C3	1.387 (4)	C11—H11A	0.93
C2—H2B	0.93	C12—C13	1.526 (4)
C3—C8	1.386 (4)	C12—H12A	0.97
C3—H3A	0.93	C12—H12B	0.97
C4—C11	1.306 (4)	C13—H13A	0.96
C4—C7	1.518 (3)	C13—H13B	0.96
C4—H4A	0.93	C13—H13C	0.96
			
C9—N1—C6	124.4 (2)	O2—C7—C4	112.9 (2)
C9—N1—C7	113.05 (18)	N1—C7—C4	100.05 (19)
C6—N1—C7	122.47 (19)	C12—C7—C4	113.7 (2)
C2—C1—C5	121.2 (2)	C10—C8—C3	121.2 (2)
C2—C1—H1A	119.4	C10—C8—H8A	119.4
C5—C1—H1A	119.4	C3—C8—H8A	119.4
C7—O2—H2A	109.5	O1—C9—N1	126.2 (2)
C1—C2—C3	119.1 (3)	O1—C9—C11	127.9 (2)
C1—C2—H2B	120.4	N1—C9—C11	105.9 (2)
C3—C2—H2B	120.4	C8—C10—C5	119.2 (2)
C8—C3—C2	120.0 (3)	C8—C10—C6	120.7 (2)
C8—C3—H3A	120	C5—C10—C6	120.0 (2)
C2—C3—H3A	120	C4—C11—C9	109.5 (2)
C11—C4—C7	111.5 (2)	C4—C11—H11A	125.3
C11—C4—H4A	124.3	C9—C11—H11A	125.3
C7—C4—H4A	124.3	C7—C12—C13	115.8 (2)
C10—C5—C1	119.2 (3)	C7—C12—H12A	108.3
C10—C5—H5A	120.4	C13—C12—H12A	108.3
C1—C5—H5A	120.4	C7—C12—H12B	108.3
N1—C6—C10	113.5 (2)	C13—C12—H12B	108.3
N1—C6—H6A	108.9	H12A—C12—H12B	107.4
C10—C6—H6A	108.9	C12—C13—H13A	109.5
N1—C6—H6B	108.9	C12—C13—H13B	109.5
C10—C6—H6B	108.9	H13A—C13—H13B	109.5
H6A—C6—H6B	107.7	C12—C13—H13C	109.5
O2—C7—N1	110.21 (19)	H13A—C13—H13C	109.5
O2—C7—C12	107.48 (19)	H13B—C13—H13C	109.5
N1—C7—C12	112.5 (2)		
			
C5—C1—C2—C3	0.8 (4)	C7—N1—C9—O1	−179.5 (2)
C1—C2—C3—C8	0.1 (4)	C6—N1—C9—C11	−176.7 (2)
C2—C1—C5—C10	−1.5 (4)	C7—N1—C9—C11	0.3 (3)
C9—N1—C6—C10	−92.1 (3)	C3—C8—C10—C5	−0.5 (4)
C7—N1—C6—C10	91.2 (3)	C3—C8—C10—C6	178.9 (2)
C9—N1—C7—O2	−119.7 (2)	C1—C5—C10—C8	1.4 (4)
C6—N1—C7—O2	57.4 (3)	C1—C5—C10—C6	−178.0 (2)
C9—N1—C7—C12	120.4 (2)	N1—C6—C10—C8	57.8 (3)
C6—N1—C7—C12	−62.5 (3)	N1—C6—C10—C5	−122.8 (2)
C9—N1—C7—C4	−0.6 (3)	C7—C4—C11—C9	−0.6 (3)
C6—N1—C7—C4	176.5 (2)	O1—C9—C11—C4	180.0 (3)
C11—C4—C7—O2	117.8 (3)	N1—C9—C11—C4	0.2 (3)
C11—C4—C7—N1	0.7 (3)	O2—C7—C12—C13	177.5 (2)
C11—C4—C7—C12	−119.4 (3)	N1—C7—C12—C13	−61.0 (3)
C2—C3—C8—C10	−0.2 (4)	C4—C7—C12—C13	51.8 (3)
C6—N1—C9—O1	3.5 (4)		

### Hydrogen-bond geometry (Å, º)

**Table:**

*D*—H···*A*	*D*—H	H···*A*	*D*···*A*	*D*—H···*A*
O2—H2*A*···O1^i^	0.82	1.95	2.772 (3)	176

Symmetry code: (i) −*x*, *y*+1/2, −*z*+2.
